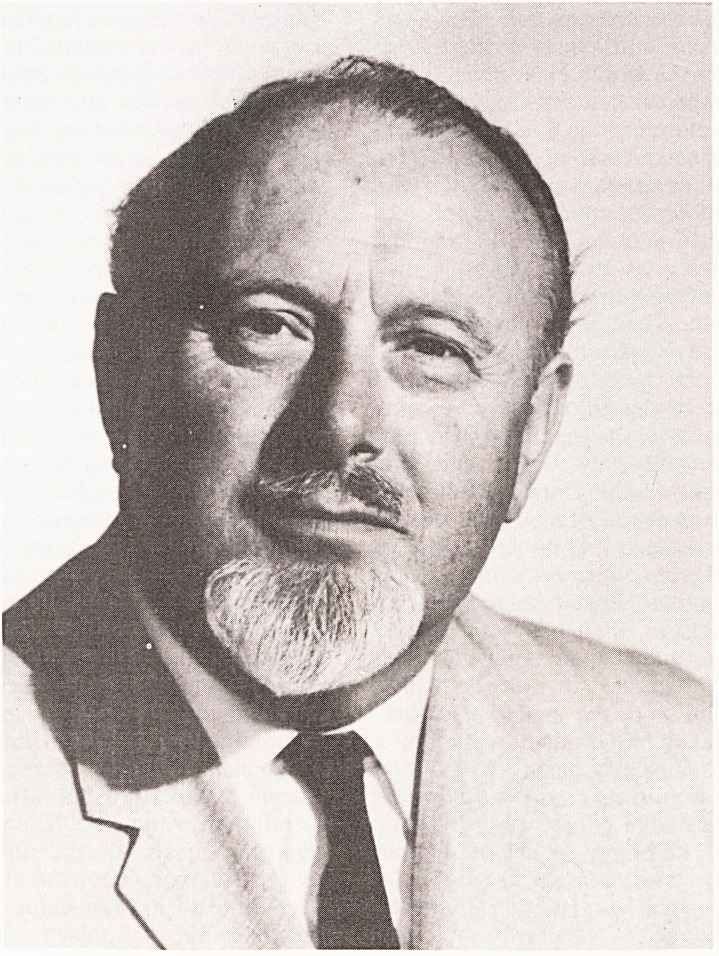# Sir Howard Middlemiss

**Published:** 1983-07

**Authors:** 


### Obituary

### Professor Sir Howard Middlemiss C.M.G., M.D., F.R.C.P., F.R.C.S., F.R.C.R.

We regret to report the sudden death of Sir Howard Middlemiss, in his 67th year on 27th April 1983. We extend our sympathy to Lady Middlemiss and their son and two daughters.

A Service of Thanksgiving for his life and work was held at the Cathedral Church of the Holy and Undivided Trinity on 10th June, and conducted by Bishop Oliver Tomkins, formerly Bishop of Bristol.

The lesson was taken from St Matthew, Chapter 5 verses 1–16 and was read by Sir Alec Merrison, Vice-Chancellor of the University of Bristol. An extract from *Only One Earth* by Dame Barbara Ward and René Dubos was read by Professor Ken Evans, University Hospital of Wales, Warden of the Royal College of Radiologists.

The following appreciation of Sir Howard's life and work was given by Professor Rhys Davies, University of Bristol.

Professor Sir Howard Middlemiss was known to all of us—that is why we are here. He was known to a cross-section of people similar to this throughout the United Kingdom, the Commonwealth and beyond. He was known to so many of them by his Christian name alone, that if I find myself referring to him by his Christian name I shall not be in the least bit dismayed.

He was known *by* his Christian name, but *for* his Christian virtues and it is because he had them in such profusion that we express our thanksgiving today.

There were clear intimations of the future during his undergraduate career at the University of Durham. Most people are content to achieve one notable event during this period of their lives. Howard achieved four that I know of. He played cricket for the University, and I am told there was hardly a club ground in the whole of Northumbria at which he had not kept wicket. He displayed his enthusiasm for travel by crossing Europe as far as Salzburg to listen to the music of Mozart—on a bicycle that had been constructed specially for him. He was identified by that most critical of all panels—his contemporary undergraduates—as the one most likely to succeed in his year. How perceptive they were. Most important of all, he met Mary Pirrie also a medical undergraduate. They were married soon after he qualified and began a partnership that displayed for more than four decades that most Christian of all virtues, service to others.

Almost immediately after he qualified, Howard joined the Royal Army Medical Corps and saw service in Normandy and in India. During this time he gained considerable experience of medicine, and of travelling abroad, and realised that he had a flair for administration. After the war he returned to Newcastle and committed himself to a career in radiodiagnosis—a decision that has had consequences more far reaching and beneficial than anyone could have imagined at that time. His Doctorate of Medicine and the necessary postgraduate qualifications put him on the threshold of a rewarding career in his chosen specialty. Bristol had the foresight to invite him to take on the first major challenge of his professional career when he was appointed Director of Radiology in 1949. He set about a major creative task, that of setting up a department that acquired a world-wide reputation. He attracted able people to work with him and inspired them, not for the only time in his life, with his own enthusiasm. He went out of his way to make contact with doctors outside his own specialty and the links that he pioneered with them have become the accepted practice, not only in Bristol but universally. Above all he showed his interest in postgraduate and in undergraduate students. His generous nature naturally included imparting all the knowledge that he had to other people and whenever he was teaching he was surrounded by people eager to learn his sage ways. He encouraged young men in the pursuit of academic excellence, and helped them to see their research through to publication. In 1966 this University recognised his contribution to medical education and academic radiology by appointing him to a Personal Chair—at that time one of only two Chairs in Radiodiagnosis in undergraduate teaching hospitals in the whole kingdom. His zealous commitment to the University included three very busy years as Dean of the Faculty of Medicine from 1977 to 1980.

While he was so energetically and successfully building up his academic unit in Bristol he had been invited—fortuitously so he always said—to visit Africa in an advisory capacity as long ago as 1953. So began an unrivalled commitment of heart and mind to the underprivileged in the developing countries. Both service and creativity were strongly evident in this unambiguous commitment. It involved literally rolling up his sleeves and dismantling equipment in order to repair it; it involved hazardous journeys to visit remote outposts; it involved flying by helicopter close to the front line in Vietnam in order to visit a hospital and conduct a ward round in French; it involved filling other people with his own enthusiasms so that they in turn visited centres in East Africa, West Africa, the West Indies and Malaysia in order to encourage a high standard of radiology and radiography and to train specific individuals and teams to carry out work that was needed; it involved arranging for whole teams to carry out work that was needed; it involved arranging for whole teams of radiographers and radiologists from these countries to visit the United Kingdom, usually in Bristol, in order to learn new skills that they could practice in their own environments. Thus, Howard was in constant demand to give advice to a whole host of countries under the aegis of the British Council and World Health Organisation. Also he travelled under the flag of the Inter University Council and was responsible for postgraduate and technician training courses in many countries. Several years ago he attended a Reception in London at which he received a large number of overseas guests. He did not have advance knowledge of everyone there but I know that he recognized each one by his full name and this was not only because he recognised each one as an individual but because he cared generously for the welfare of each one. Recently he found that one of his friends, a radiographer from Vietnam, had escaped and was in a transit camp with very little hope. Howard initiated an interest that culminated in the ultimate arrival of his friend in a free country. The delight that he displayed at that time was characteristic of the overt pleasure that he showed in the success of others.

This interest in the success of others was not a new thing and much earlier in his career it had led logically to the wish to provide facilities for training and education. He and many of his contemporaries believed passionately in the establishment of a Royal College of Radiologists. He had been involved energetically with its predecessor, the Faculty of

Radiologists, for nearly a quarter of a century. When he became President of the Faculty he launched, from the platform created by his predecessors, a final sustained and successful effort, in a unique combination with Lord Robens. Their efforts culminated in the granting of a Supplemental Charter by the Privy Council in 1975. He told me that it was one of the happiest days of his life and he was characteristically working in the West Indies when he heard the news. He became the first President of the Royal College of Radiologists and filled the office with his own style and dignity. When his term of office was over he continued the energetic interest he had in International Education and in setting up an examination structure used in all the European countries.

Service and success were their own rewards for Howard but also he received numerous honours. He was justifiably and immensely proud of the Companionship of the Order of St Michael and St George that was awarded to him for his services overseas. Fellowships of Colleges and international associations were showered upon him. He was invited to give no fewer than nine named lectures in this country and abroad. When the honour of a Knighthood was conferred upon him in 1981 for services to radiology, international radiology rejoiced and he was the first to say that the honour was to radiology. In 1982 he was awarded the Gold Medal of the Royal College of Radiologists. This is the highest honour the College can give and it is awarded for outstanding contribution to the development and teaching of radiology. It is given rarely, and was richly deserved by this man of fine intellect and liberal outlook.

We are reminded thus of his unswerving loyalty to causes that he believed in, his service to others, his creative achievements and the honours that his peers conferred upon him. These are the attributes of a very special man, a man who loved his home in Bristol, who opened it generously to literally scores of students from the whole Commonwealth and beyond and who took delight and pleasure in their friendship and success. He was full of creative energy and dedicated to conservation of resources. In recent years he and Mary had tackled a small neglected woodland nestling in the Malverns. Their first move was to cut bold pathways through it and at this stage they invited colleagues and their families to visit them there. Howard allowed the children to roam along the paths he had cut in this woodland, but only after he had armed each one of them with a piercing whistle and instructions to blow it if they were lost. When the deadwood had been cut and carried away he began to replant it with oak trees, an act that we can perhaps think of now as being symbolic of his whole life. Howard's life was indeed one of promise fulfilled, and there is much for us to rejoice in having known him. His presence graced this University and this City, as well as radiology and its Royal College, and he inspired a whole generation of students and enriched the life of everyone who met him.

Our thanksgiving is for his life and work and for the privilege of knowing him, shared by us and so many others."

## Figures and Tables

**Figure f1:**